# Impact of Opioid and Cannabis Use on Low‐Dose Amitriptyline Efficacy in Cyclical Vomiting Syndrome: A Real‐World Study in the United Kingdom

**DOI:** 10.1111/nmo.70007

**Published:** 2025-02-27

**Authors:** Mohsin F. Butt, Francesca Cefalo, Caterina Sbarigia, Arkadeep Dhali, Maura Corsetti

**Affiliations:** ^1^ NIHR Nottingham Biomedical Research Centre Nottingham University Hospitals NHS Trust and the University of Nottingham Nottingham UK; ^2^ Nottingham Digestive Diseases Centre, Translational Medical Sciences, School of Medicine University of Nottingham Nottingham UK; ^3^ Academic Department of Gastroenterology Sheffield Teaching Hospitals NHS Foundation Trust Sheffield UK

**Keywords:** cyclic vomiting syndrome, disorder of gut‐brain interaction, functional gastrointestinal disorder, opioids

## Abstract

**Background:**

Central neuromodulators, specifically tricyclic antidepressants (TCAs), are prescribed as prophylactic treatment for cyclical vomiting syndrome (CVS). It is unclear whether opioids and/or cannabis affect the treatment response to neuromodulators. The aims of this study were to assess: (i) the prevalence of opioid and cannabis use among outpatients with CVS, (ii) clinical characteristics associated with opioid/cannabis use and response to a three‐tiered neuromodulator treatment algorithm, and (iii) the effect of opioid/cannabis cessation on response to the treatment algorithm.

**Methodology:**

Data from consecutive patients newly diagnosed with Rome IV CVS at a single tertiary care neurogastroenterology outpatient clinic (January 2016–June 2024) were retrospectively collected. Patients were advised to stop consuming opioids and/or cannabis and commenced a low‐dose TCA.

**Results:**

Sixty‐one (46/75) percent of outpatients with CVS responded to the three‐tiered treatment algorithm. Among responders, 42 (91%) patients responded to TCA alone (1st line therapy), 3 (7%) patients responded to TCA and selective serotonin reuptake inhibitor or serotonin norepinephrine reuptake inhibitor (2nd line therapy), and 1 (2%) patient required topiramate (3rd line therapy). The mean [SD] dosage of TCA among responders was 26.5 [18.3] mg. Twenty‐five (33%) patients consumed opioids, 14 (19%) took cannabis, and five (7%) consumed both opioids and cannabis. While opioid cessation was associated with clinical response to the treatment algorithm (*p* = 0.03), opioid intake at the initial consultation was not (*p* = 0.2). Irritable bowel syndrome was independently associated with significantly greater odds (OR [95% CI]) of opioid consumption at baseline (6.59 [1.49–29.24], *p* = 0.01). Heartburn was independently associated with lower odds of response to the treatment algorithm (0.2 [0.05–0.65], *p* = 0.006).

**Conclusion:**

Low‐dose neuromodulators, along with opioid and cannabis cessation, may be important strategies in the management of CVS.

## Graphical abstract

This article presents the outcomes of a three‐tiered outpatient treatment algorithm for the prophylactic management of cyclical vomiting syndrome (CVS). Three key messages: (1) 61% of patients responded to the treatment algorithm, (2) opioid cessation was associated with clinical response to the treatment algorithm (*p* = 0.03), and (3) a low‐dose tricyclic antidepressant (mean [SD] dosage: 26.5 [18.3] mg) may be effective in the prophylactic management of CVS.

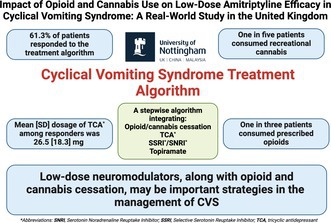

**Summary**

- Sixty‐one percent of patients with cyclical vomiting syndrome (CVS) responded to a three‐tiered prophylactic treatment algorithm.
- Opioid cessation was associated with clinical response to the treatment algorithm (*p* = 0.03).
- A low‐dose tricyclic antidepressant (mean [SD] dosage 26.5 [18.3] mg) may be effective in the prophylactic management of CVS.

## 1 Introduction

Cyclical vomiting syndrome (CVS), a disorder of gut–brain interaction (DGBI) characterized by stereotypical episodes of nausea and vomiting [1], affects up to 2% of people in the general population of the United Kingdom (UK) and North America [2]. The etiology of CVS is multifactorial and several genetic [3, 4], environmental (e.g., exposure to cannabis [5]), autonomic [6, 7], and neurohormonal [8] mechanisms are thought to contribute to the genesis and evolution of the disease. CVS can significantly impair quality of life [9, 10, 11] and is associated with substantial personal and societal economic costs [12, 13], including those related to emergency department visits [14].

CVS should be managed using the biopsychosocial approach [15] whereby lifestyle modifications (e.g., trigger avoidance) should be integrated with evidence‐based psychological therapy and prophylactic and/or abortive medications [16]. North American Clinical Guidelines emphasize the role of amitriptyline, a tricyclic antidepressant (TCA), as the first‐line prophylactic therapy for CVS [16]. The therapeutic dose of TCA in the management of CVS has yet to be confirmed in randomized controlled trial settings but is reported to range between 75 and 100 mg daily [17, 18].

Chronic opioid use has been reported by 23% [17] to 28.6% [19] of patients with CVS, oftentimes for the management of concurrent abdominal pain [20], and is associated with higher rates of hospitalizations [19, 21] and non‐response to TCA therapy [22]. The moderately high prevalence of opioid consumption persists despite limited evidence supporting narcotic use in the management of non‐malignant chronic pain [23], their known association with dependence, as well as adverse gastrointestinal (GI) side‐effects [24]. Cannabis, consumed by up to 68% of patients with CVS for its potential anxiolytic and anti‐emetic properties [5], may reduce the efficacy of prophylactic TCA [22].

The University of Nottingham has developed a three‐tiered outpatient treatment algorithm for the prophylactic management of CVS: (i) opioid/cannabis cessation in conjunction with a low‐dose (5 mg) TCA, up‐titrated to clinical remission; (ii) the addition of a selective serotonin reuptake inhibitor (SSRI)/serotonin norepinephrine reuptake inhibitor (SNRI); and (iii) topiramate following TCA/SSRI/SNRI cessation.

The aims of this retrospective study were to determine: (i) the prevalence of opioid and cannabis use among outpatients diagnosed with CVS in a UK neurogastroenterology referral center, (ii) clinical characteristics associated with opioid/cannabis use and response to the three‐tiered neuromodulator treatment algorithm, and (iii) whether adherence to opioid and cannabis cessation advice affected clinical response to the treatment algorithm.

## 2 Methodology

### 2.1 Setting

Data were retrospectively collected from consecutive adults (aged ≥ 18 years) diagnosed with Rome IV CVS [1] in a tertiary care neurogastroenterology outpatient clinic (Queen's Medical Centre, Nottinghamshire, UK) between January 2016 and June 2024. This study was approved as an audit by Nottingham University Hospitals NHS Trust (reference: 24‐006C).

### 2.2 Data Capture

A standardized clinic template was used to collect data related to: age, sex, GI symptoms (abdominal pain, diarrhea, constipation, alternating bowel movements, early satiety, post‐prandial fullness, heartburn, and dysphagia), concomitant DGBI (irritable bowel syndrome [IBS], functional dyspepsia, functional constipation, functional diarrhea, other DGBI), psychological co‐morbidities, chronic pain diagnoses (primary headache disorder, fibromyalgia, lower back pain), previous abdominal/pelvic surgery, past and current medication history (specifically TCA, opioid, SSRI, SNRI, benzodiazepine, anti‐emetics, beta blocker, and/or triptan use), and investigations (endoscopic procedures, computed tomography [CT] of the GI tract, magnetic resonance imaging [MRI] of the GI tract, ultrasound of the GI tract, high resolution esophageal manometry, and anorectal manometry).

Only those patients newly diagnosed with CVS by the senior author (MC) in a single neurogastroenterology clinic were studied. To ensure reliable and accurate data acquisition, patient information recorded in consultation notes was corroborated with medical records. Data were independently collected by two clinicians (CS and FC), and any discrepancies were resolved through consensus by MFB and MC.

### 2.3 Nottingham CVS Treatment Protocol

**FIGURE 1:**
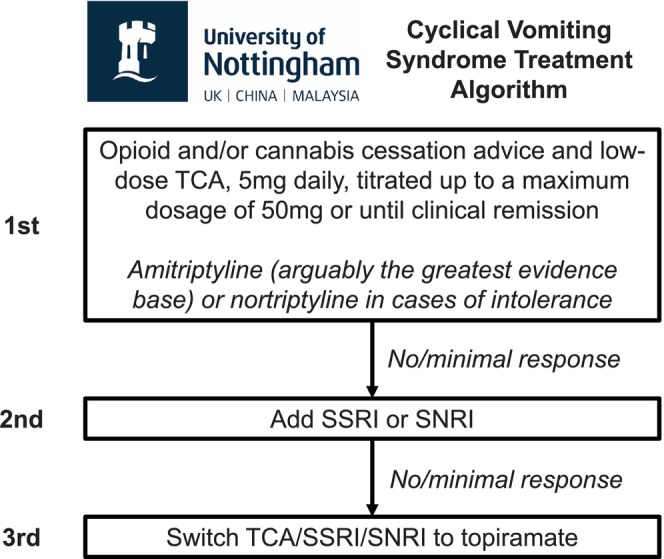
The University of Nottingham treatment algorithm for the prophylactic management of CVS in the outpatient setting. CVS, cyclical vomiting syndrome; SNRI, serotonin‐norepinephrine reuptake inhibitor; SSRI, selective serotonin reuptake inhibitor; TCA, tricyclic antidepressant.

Figure 1 describes the three‐tiered University of Nottingham treatment algorithm for the prophylactic management of CVS in the outpatient setting. All patients who were newly diagnosed with CVS and were not taking a TCA were prescribed amitriptyline. TCAs were prescribed if patients consented to treatment and had no medical contraindications.

Patients were prescribed 5 mg amitriptyline, which was increased to 10 mg after 10–20 days, if tolerated, which was reviewed at a follow‐up outpatient consultation. At follow‐up consultations (once every 3 months), the TCA dosage was up‐titrated until clinical remission was achieved, defined as the absence of stereotypical episodes of vomiting for at least 6 months, or the maximum dosage (50 mg daily) was prescribed.

Patients were prescribed a TCA in combination with an SSRI if they had no or minimal response to TCA therapy alone, in line with the augmentation approach recommended by the Rome Foundation [25]. Patients who required combination therapy (TCA and SSRI) were prescribed 5 mg of SSRI for 10–20 days, which was up‐titrated to 10 mg, if tolerated, and reviewed at a 3 month follow‐up outpatient consultation. The SSRI dosage was up‐titrated until clinical remission was achieved, or the maximum dose was prescribed. In cases where patients had no or minimal response to the TCA and SSRI combination therapy, the SSRI was switched to an SNRI, such as duloxetine, at a starting dosage of 20 mg. Duloxetine was up‐titrated until clinical remission was achieved, or the maximum dosage (120 mg daily) was prescribed. Where patients reported no or minimal response to the TCA/SSRI/SNRI combination therapy, they were switched to topirimate, which was considered third‐line therapy [16, 26].

### 2.4 Opioid and Cannabis Cessation Advice

Patients who consumed opioids for chronic non‐malignant pain were provided a verbal explanation about the side effects of narcotic use and the benefits of opioid cessation. Additionally, patients were directed to online resources and shown testimonies from patients who had successfully stopped taking opioids.

Consistent with recommendations from the Centers for Disease Control and Prevention [27], the total opioid dosage was typically tapered by 10% per month if patients consumed opioids for more than a year. For patients who had consumed opioids for a shorter duration of time (weeks to months), they may have been able to tolerate a faster down‐titration of up to 10% per week. Adherence to opioid cessation advice was assessed by self‐reported measures.

Long‐term cannabis use has been associated with cannabinoid hyperemesis syndrome (CHS), which shares clinical similarities with CVS [5]. Hence, patients were advised to discontinue cannabis due to its potential to exacerbate CVS symptoms. Cannabis is classified as a Class B drug under the UK Misuse of Drugs Act 1971, which denotes it as an illicit substance with strict regulations regarding possession, cultivation, and distribution. Therefore, recommendations to cease cannabis consumption were in line with UK law.

Patients who did not respond to the treatment algorithm were consented to undergo urine toxicology screening to confirm cessation of opioid and cannabis use (defined as complete discontinuation of cannabis and/or opioid intake since the previous outpatient consultation), consistent with UK Drug Misuse and Dependence Guidelines [28].

### 2.5 Statistical Analysis

Continuous and categorical variables were expressed as mean ± standard deviation and number (%), respectively. A comparison of continuous variables was performed using the unpaired t‐test and the Fisher's exact test for categorical variables. Based on domain expertise and outcomes from the univariate logistic regression models, two separate multiple logistic regression models were created to assess factors that were associated with (i) clinical response to the treatment algorithm and (ii) opioid consumption at baseline.

The following predictor variables were used in the multivariate logistic regression model to identify clinical characteristics associated with clinical response to the treatment algorithm: (i) age, (ii) sex, (iii) heartburn, (iv) opioid consumption at initial consultation, (v) anti‐emetics (5‐HT3/D2/histamine receptor antagonist), and (vi) cannabis use.

The following predictor variables were used in the multivariate logistic regression model to identify clinical characteristics associated with opioid consumption at the initial consultation: (i) age, (ii) sex, (iii) previous abdominal/pelvic surgery, (iv) chronic pain diagnosis (primary headache disorder, fibromyalgia, lower back pain), (v) depression and/or anxiety, and (vi) IBS.

For all tests, a two‐sided *p* value of < 0.05 was considered significant. All statistical computations were performed using JMP (SAS Institute).

## 3 Results

### 3.1 Clinical Response to Treatment Algorithm

**FIGURE 2:**
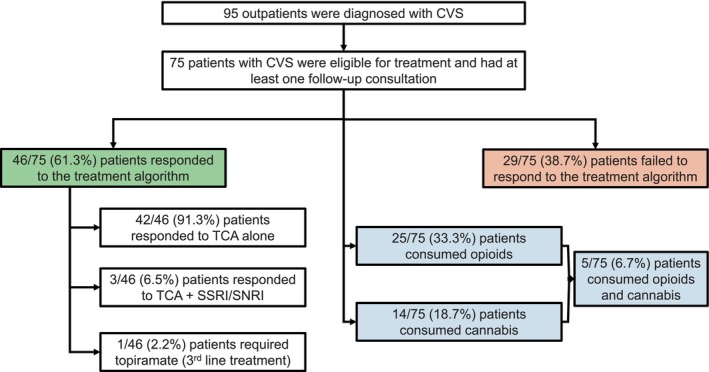
A flowchart illustrating the proportion of patients with CVS who responded to the treatment algorithm and were consuming prescribed opioids and/or cannabis. CVS, cyclical vomiting syndrome; SNRI, serotonin norepinephrine reuptake inhibitor; SSRI, selective serotonin reuptake inhibitor; TCA, tricyclic antidepressant.

Ninety‐five outpatients were newly diagnosed with CVS (Figure 2). Among the 75 outpatients who were seen on at least two occasions (mean [SD] duration of follow‐up: 29.4 [20.2] months), 46 (61.3%) responded to the treatment algorithm. Patients who responded to the treatment algorithm were followed up for a longer duration than non‐responders (36.1 [22.2] months vs. 24.8 months [17.6], *p* = 0.02).

Among the 46 responders, 42 (91.3%) patients responded to TCA alone, 3 (6.5%) patients responded to TCA and SSRI/SNRI, and 1 (2.2%) patient required topiramate (Figure 2). Only three (4.0%) patients were intolerant to TCA treatment. The mean [SD] dosage of amitriptyline (26.5 [18.3] vs. 23.8 [17.3] mg, *p* = 0.6) did not differ between responders and non‐responders.

**FIGURE 3:**
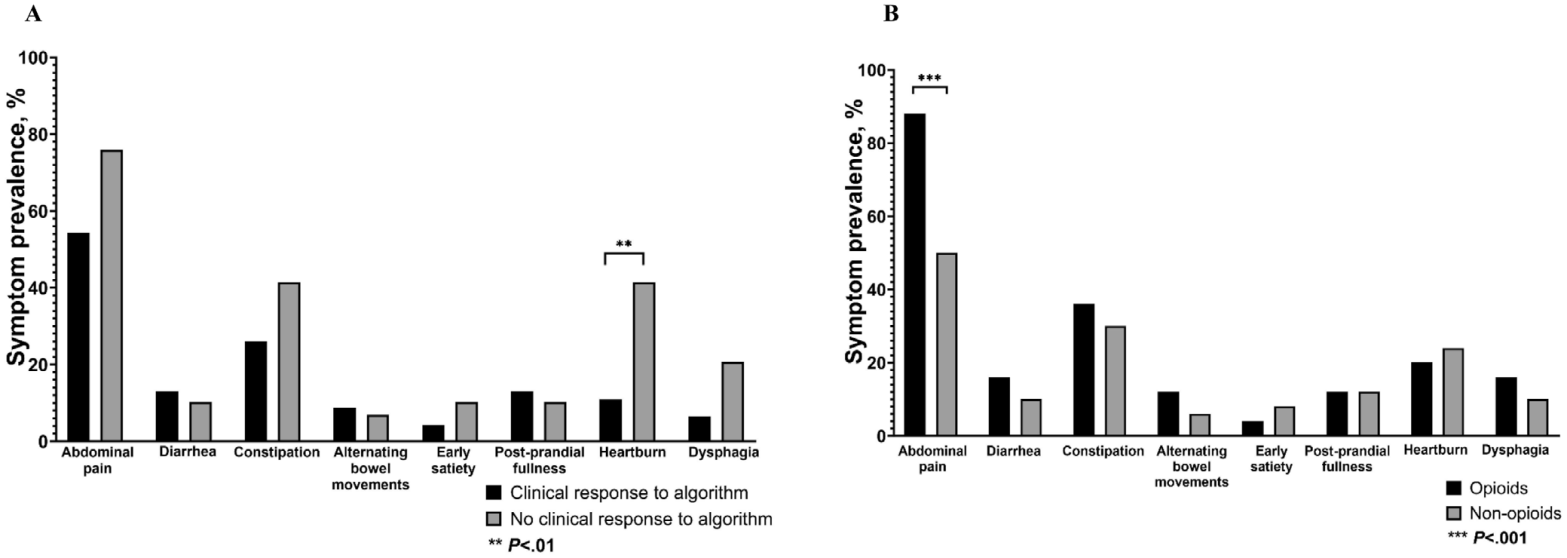
Gastrointestinal symptoms reported by patients at the initial consultation who (3A) responded to the treatment algorithm and (3B) were consuming prescribed opioids.

Among responders versus non‐responders to the treatment algorithm, there was no difference in the proportion of patients who reported cannabis use (13.0% vs. 27.6%, *p* = 0.1) or were taking prescribed opioids at the initial consultation (28.2% vs. 41.4%, *p* = 0.2) (Table 1). A greater proportion of responders were taking anti‐emetics versus non‐responders (67.4% vs. 37.9%, *p* = 0.01) (Table 1). There was no difference in the mean number of concomitant DGBI among responders versus non‐responders (0.33 [0.60] vs. 0.59 [0.68], *p* = 0.09) (Table 1). Regarding individual GI symptoms, a greater proportion of non‐responders reported heartburn versus responders (41.4% vs. 10.9%, *p* = 0.002) (Figure 3A). In a multivariate logistic regression model, heartburn was associated with significantly lower odds [95% CI] of responding to the treatment algorithm (0.2 [0.05–0.65], *p* = 0.006) (Table 2).

**TABLE 1:** Differences in demographics and medical history according to clinical response to the treatment algorithm.

Patient characteristic	Clinical response to the treatment algorithm (*n* = 46)	No clinical response to the treatment algorithm (*n* = 29)	*p* ^a^	OR (95% CI) for clinical response to the treatment algorithm^b^	*p* ^c^
Demographics					
Sex, Female, *n* (%)	38 (82.6%)	19 (65.5%)	0.09	2.5 (0.85–7.37)	0.09
Age, years, mean (SD)	30.48 (12.93)	32.31 (9.45)	0.5	0.99 (0.95–1.03)	0.5
Ethnicity, Caucasian, *n* (%)	31 (67.4%)	18 (62.0%)	0.7	1.35 (0.32–5.90)	0.7
Concomitant DGBI					
Concomitant DGBI, mean (SD)	0.33 (0.60)	0.59 (0.68)	0.09	0.50 (0.23–1.09)	0.08
Irritable bowel syndrome, *n (%)*	6 (13.0%)	9 (31.0%)	0.06	0.33 (0.10–1.07)	0.06
Functional dyspepsia, *n (%)*	3 (6.5%)	2 (6.9%)	0.9	0.94 (0.15–6.00)	0.9
Functional constipation, *n (%)*	3 (6.5%)	1 (3.4%)	0.5	1.95 (0.19–19.73)	0.6
Other DGBI, *n (%)*	3 (6.5%)	5 (17.2%)	0.1	0.33 (0.07–1.53)	0.2
Non‐gastrointestinal co‐morbidities					
Depression and/or anxiety, *n* (%)	13 (28.3%)	11 (37.9%)	0.4	0.64 (0.24–1.73)	0.4
Chronic pain (primary headache disorder, fibromyalgia, lower back pain), *n* (%)	11 (23.9%)	7 (24.1%)	> 0.99	0.99 (0.33–2.93)	> 0.99
Previous abdominal surgery, *n* (%)	10 (21.7%)	12 (41.4%)	0.07	0.39 (0.14–1.09)	0.07
Cannabis use, *n* (%)	6 (13.0%)	8 (27.6%)	0.1	0.39 (0.12–1.29)	0.1
Medications and healthcare resource utilization prior to the first consultation					
Previous TCA prescription, *n* (%)	16 (34.8%)	11 (37.9%)	0.8	0.87 (0.33–2.29)	0.8
Opioid prescription, *n* (%)	13 (28.2%)	12 (41.4%)	0.2	0.56 (0.21–1.49)	0.2
SSRI and/or SNRI prescription, *n* (%)	15 (32.6%)	8 (27.6%)	0.9	1.08 (0.40–2.92)	0.9
Benzodiazepine, *n* (%)	5 (10.9%)	5 (17.2%)	0.4	0.56 (0.15–2.14)	0.4
Anti‐emetic (5‐HT3R/D2R/H1R antagonist), *n* (%)	31 (67.4%)	11 (37.9%)	0.01	3.38 (1.28–8.9)	0.01
Beta blocker, *n* (%)	7 (15.2%)	2 (6.9%)	0.3	2.42 (0.47–12.57)	0.3
Triptan, *n* (%)	3 (6.5%)	0 (0%)	0.08	#	#
Gastrointestinal investigations prior to the first consultation, mean (SD)	1.76 (1.16)	2.10 (1.37)	0.3	0.80 (0.55–1.17)	0.2

Abbreviations: 5‐HT3, 5‐hydroxytryptamine type‐3 receptor; D2, dopamine type‐2 receptor; DGBI, disorder of gut–brain interaction; H1R, histamine type‐1 receptor; SNRI, serotonin norepinephrine reuptake inhibitor; SSRI, selective serotonin reuptake inhibitor; TCA, tricyclic antidepressant.

# Regression cannot be performed when there are no variables in a group.

^a^ *p* value for unpaired *t*‐test (continuous variables) or Fisher's exact test (categorical variables).

^b^ Univariate regression.

^c^ *p* value for univariate regression.

**TABLE 2:** A multivariate logistic regression model to identify factors associated with clinical response to the treatment algorithm.

Patient characteristic	Clinical response to the treatment algorithm (*n* = 46)	No clinical response to the treatment algorithm (*n* = 29)	OR (95% CI) for clinical response to the treatment algorithm	*p*
Age, years, mean (SD)	30.48 (12.93)	32.31 (9.45)	0.99 (0.94–1.03)	0.6
Sex, Female, *n* (%)	38 (82.6%)	19 (65.5%)	2.05 (0.57–7.36)	0.3
Heartburn, *n* (%)	5 (10.9%)	12 (41.4%)	0.2 (0.05–0.65)	0.006
Opioid prescription at the initial consultation, *n* (%)	13 (28.2%)	12 (41.4%)	0.44 (0.14–1.40)	0.2
Anti‐emetics (5‐HT3R/D2R/H1R antagonist), *n* (%)	31 (67.4%)	11 (37.9%)	2.75 (0.93–8.11)	0.07
Cannabis use, *n* (%)	6 (13.0%)	8 (27.6%)	0.5 (0.13–1.94)	0.3

Abbreviations: 5‐HT3, 5‐hydroxytryptamine type‐3 receptor; D2R, dopamine type‐2 receptor; H1R, histamine type‐1 receptor.

### 3.2 Opioid and Cannabis Use

Among the 75 outpatients who were seen on at least two occasions, 25 (33.3%), 14 (18.7%), and 5 (6.7%) patients were consuming opioids, cannabis, and opioids and cannabis, respectively (Figure 2).

Patients who consumed opioids had a higher mean [SD] number of concomitant DGBI versus non‐users (0.7 [0.7] vs. 0.3 [0.5], *p* = 0.02), specifically IBS (36% vs. 12%, *p* = 0.02) (Table 3). Regarding individual GI symptoms, a greater proportion of patients who consumed opioids reported abdominal pain versus non‐users (88.0% vs. 50.0%, *p* < 0.001) (Figure 3B). In a multivariate logistic regression model, IBS was independently associated with greater odds (OR [95% CI]: 6.59 [1.49–29.24], *p* = 0.01) of opioid consumption at baseline (Table 4).

**TABLE 3:** Differences in demographics and medical history between patients who consumed opioids at baseline versus non‐users.

Patient characteristic	Opioids (*n* = 25)	Non‐opioid (*n* = 50)	*p* ^a^	OR (95% CI) for opioid consumption at baseline^b^	*p* ^c^
Demographics					
Sex, Female, *n* (%)	21 (84.0%)	36 (72.0%)	0.2	2.04 (0.59–7.01)	0.3
Age, years, mean (SD)	34.2 (11.5)	29.7 (11.6)	0.1	1.03 (0.99–1.08)	0.1
Ethnicity, Caucasian, *n* (%)	16 (64.0%)	33 (66.0%)	0.3	0.48 (0.12–1.92)	0.3
Concomitant DGBI					
Comorbid DGBI, mean (SD)	0.7 (0.7)	0.3 (0.5)	0.02	2.69 (1.20–6.00)	0.01
Irritable bowel syndrome, *n (%)*	9 (36%)	6 (12%)	0.02	4.12 (1.27–13.44)	0.02
Functional dyspepsia, *n (%)*	3 (12.0%)	2 (4.0%)	0.2	3.27 (0.51–21.00)	0.2
Functional constipation, *n (%)*	2 (8.0%)	2 (4.0%)	0.5	2.09 (0.28–15.76)	0.5
Other DGBI, *n (%)*	3 (12.0%)	5 (10.0%)	0.8	1.23 (0.27–5.61)	0.8
Non‐gastrointestinal co‐morbidities					
Depression and/or anxiety, *n* (%)	11 (44.0%)	13 (26.0%)	0.1	2.24 (0.81–6.15)	0.1
Chronic pain (primary headache disorder, fibromyalgia, lower back pain), *n* (%)	9 (36.0%)	9 (18.0%)	0.09	2.56 (0.86–7.62)	0.09
Previous abdominal/pelvic surgery, *n* (%)	11 (44.0%)	11 (22.0%)	0.05	2.79 (0.99–7.84)	0.05
Cannabis abuse, *n* (%)	5 (20.0%)	9 (18.0%)	0.8	1.14 (0.3–3.85)	0.8
Medications and healthcare resource utilization prior to the first consultation					
Previous TCA prescription, *n* (%)	10 (40.0%)	17 (34.0%)	0.6	1.29 (0.48–3.49)	0.6
SSRI and/or SNRI prescription, *n* (%)	10 (40.0%)	14 (28.0%)	0.3	1.71 (0.62–4.71)	0.3
Benzodiazepine, *n* (%)	5 (20.0%)	5 (10.0%)	0.3	2.2 (0.57–8.47)	0.3
Anti‐emetic (5‐HT3R/D2R/H1R antagonist), *n* (%)	14 (56.0%)	28 (56.0%)	> 0.99	1.00 (0.38–2.63)	> 0.99
Beta blocker, *n* (%)	4 (16.0%)	5 (10.0%)	0.5	1.71 (0.42–7.04)	0.5
Triptan, *n* (%)	1 (4.0%)	2 (4.0%)	> 0.99	1.00 (0.09–11.59)	> 0.99
Gastrointestinal investigations prior to the first consultation, mean (SD)	1.8 (1)	1.94 (1.36)	0.6	0.91 (0.61–1.35)	0.6

Abbreviations: 5‐HT3, 5‐hydroxytryptamine type‐3 receptor; D2, dopamine type‐2 receptor; DGBI, disorders of gut‐brain interaction; H1R, histamine type‐1 receptor; SNRI, serotonin norepinephrine reuptake inhibitor; SSRI, selective serotonin reuptake inhibitor; TCA, tricyclic antidepressant.

# Regression cannot be performed when there are no variables in a group.

^a^ *p* Value for unpaired *t*‐test (continuous variables) or Fisher's exact test (categorical variables).

^b^ Univariate regression.

^c^ *p* Value for univariate regression.

**TABLE 4:** A multivariate logistic regression model to identify factors associated with opioid consumption at baseline.

Patient characteristic	Opioids (*n* = 25)	Non‐opioid (*n* = 50)	OR (95% CI) for opioid consumption at baseline	*p*
Age, years, mean (SD)	34.16 (11.51)	29.7 (11.57)	1.02 (0.97–1.07)	0.4
Sex, Female, *n* (%)	21 (84.0%)	36 (72.0%)	3.38 (0.70–16.50)	0.1
Previous abdominal/pelvic surgery, *n* (%)	11 (44.0%)	11 (22.0%)	2.10 (0.57–7.75)	0.3
Chronic pain (primary headache disorder, fibromyalgia, lower back pain), *n* (%)	9 (36.0%)	9 (18.0%)	2.26 (0.62–8.26)	0.2
Depression and/or anxiety, *n* (%)	11 (44.0%)	13 (26.0%)	1.67 (0.54–5.20)	0.4
Irritable bowel syndrome, *n* (%)	9 (36%)	6 (12%)	6.59 (1.49–29.24)	0.01

### 3.3 Opioid and Cannabis Cessation

**FIGURE 4:**
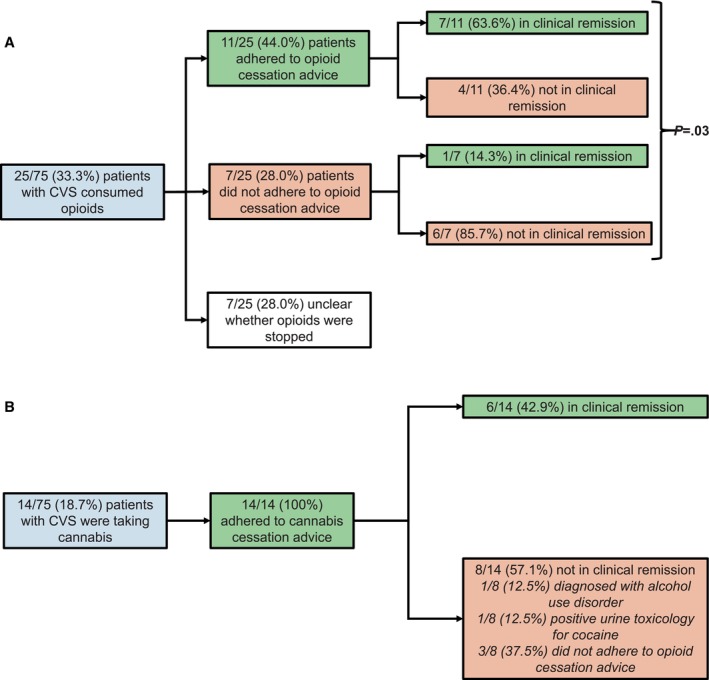
Flowcharts illustrating the impact of (4A) opioid and (4B) cannabis cessation on the response to the treatment algorithm.

Among the 25 patients consuming opioids, 11 (44%) adhered to opioid cessation advice, 7 (28%) did not adhere to opioid cessation advice, and the adherence status was unclear for 7 (28%) patients (Figure 4A). Among the patients for whom data on adherence were available (*n*=18), opioid cessation was associated with clinical response to the treatment algorithm (*p* = 0.03). Among four patients who self‐reported adherence to opioid cessation but were not in clinical remission, urine toxicology revealed that one tested positive for cannabis and another for a combination of opioids, cannabis, and cocaine.

Among the 14 patients with CVS who consumed cannabis, 100% adhered to cannabis cessation advice, among whom six (42.9%) were in clinical remission at follow‐up. Among the eight patients who were not in clinical remission after adhering to cannabis cessation advice, one (12.5%) was diagnosed with alcohol use disorder, one (12.5%) tested positive for cocaine on urine toxicology, and three (37.5%) patients did not adhere to opioid cessation advice (Figure 4B).

## 4 Discussion

The response rate to prophylactic TCA in our study (46/75, 61.3%) is broadly consistent with the 70% response rate reported in the literature [16, 18, 22, 29, 30]. Intolerance to TCA therapy was only reported in three (4%) patients, which is significantly lower than the 26%–34% intolerance rate reported at mean doses of 75–100 mg [17, 18]. These results from a single center in the UK suggest that the therapeutic effect of TCA may be achieved at a lower dosage than that prescribed in North America (75–100 mg daily) with a potentially more favorable side‐effect profile [16]. The response rate observed at a lower dosage among patients with CVS in the UK compared to those in North America might be explained by differences in symptom severity and/or genetic factors that influence neuromodulator metabolism [29].

Seventeen (22.7%) patients in our study reported heartburn, consistent with previous research demonstrating a 19% prevalence of gastroesophageal reflux disease among individuals with CVS [31]. In the multivariate logistic regression model, heartburn was independently associated with a lack of response to the treatment algorithm. It is tempting to hypothesize that patients with concomitant heartburn have a greater degree of visceral hypersensitivity, suggestive of a more dysregulated brain–gut axis, which would necessitate a potentially higher TCA dosage or combination therapy with nonpharmacological agents.

Perhaps unsurprisingly, an anti‐emetic prescription was associated with an increased odds of response to the treatment algorithm. Although this did not reach statistical significance in the multivariate logistic regression model (*p* = 0.07), this finding still implies that anti‐emetics positively influenced the effectiveness of the treatment algorithm. Therefore, whether this treatment algorithm reduces patients' consumption of abortive agents (i.e., anti‐emetics) should be evaluated in future studies.

Thirty‐three percent of patients in our study were taking opioids, consistent with prevalence rates of 23% [17] and 28.6% [19] documented in the literature. Opioids are associated with nausea and vomiting [32], so it is perhaps not surprising that opioid consumption decreased the odds of clinical response to the treatment algorithm in the multivariate logistic regression model—the lack of statistical significance may be explained by underpowering. A previous study suggests that coexisting opioid use may be associated with lack of response to TCA therapy in the prophylactic management of CVS [22].

Nineteen percent of patients with CVS in our study consumed cannabis. The higher prevalence rate of cannabis use in North American settings (37% [33] and 39% [29]) may be explained by the fact that cannabis remains illegal for recreational use in the UK, unlike in some regions of the United States. Since recreational cannabis consumption is illegal in the UK, it is possible that some patients may not have been forthcoming about usage and that the prevalence rate could have been higher had urine toxicology been performed to confirm consumption. In the only UK study of CVS (*n* = 17), cannabis use was reported in five (29%) individuals [34]. In our study, cannabis use did not impact clinical response to the treatment algorithm on multivariate analysis (*p* = 0.3). Among the patients (*n* = 14) who reported current cannabis use, all self‐reported adherence to cannabis cessation advice, among whom six (42.9%) were in clinical remission at follow‐up. Other centers have reported less success with cannabis cessation—in one case series, 70% of patients with CVS adhered to cannabis cessation recommendations, among whom 86% were in remission [35].

In our center, patients who do not achieve a clinical response to the treatment algorithm despite cannabis and/or opioid cessation are routinely consented to undergo urine drug screening. In two such cases, patients were identified to have a positive urine screening for cocaine. This finding does not imply that clinicians should perform urine toxicology on *all* patients with CVS. Instead, it suggests that urine toxicology may be warranted in select cases, particularly those involving opioid dependence or the presence of other risk factors for illicit drug use [36].

Strengths of this study relate to its large sample size (*n* = 75) of well‐characterized patients with Rome IV CVS, which builds upon the findings of a case series conducted in the UK [34]. The relatively prolonged follow‐up (29.4 [20.2] months) also accounts for the episodic nature of CVS, allowing for the consideration of symptom variation that may occur during specific times of the year (e.g., secondary to psychosocial stressors).

Our study is not without limitations, particularly those intrinsic to any retrospective analysis which employs chart review and uncontrolled medication usage. Since data were collected in routine clinical practice, patients did not complete validated GI symptom questionnaires, so the degree of change in the frequency and/or intensity of GI symptoms cannot be quantified with absolute accuracy. To address this concern, two independent clinicians (CS and FC) reviewed patient charts, and discrepancies were resolved by the first (MFB) and senior author (MC). Given patients were drawn from one consultant in a single‐center setting, the characteristics of our sample may not be entirely representative of the CVS population on a national level. However, although patients in our study were consulted in a tertiary referral center, the majority (91.3%) of patients who responded to the treatment algorithm required first‐line treatment alone, which suggests that the severity of CVS may parallel that seen in non‐referral settings.

In addition to using validated questionnaires, future studies would benefit from studying the original indication for the opioid prescription. Whether this treatment algorithm reduces reliance on the number of abortive therapies (i.e., anti‐emetics), the frequency of CVS episodes, and the duration of the emetic phase is also worthy of further evaluation. Strategies to optimize continued outpatient engagement in treatment should be explored in the future. Indeed, non‐responders may have benefited from a higher TCA dosage, but from clinical experience, many in this group had infrequently engaged with the outpatient treatment plan, which likely accounts for the shorter follow‐up duration among non‐responders versus responders (36.1 vs. 24.8 months). Finally, compared to TCAs, there is a relative paucity of data addressing SNRIs, SSRIs, and topiramate in the prophylactic management of nausea and vomiting disorders [16, 25], so this single‐center study should encourage clinicians to integrate non‐TCA alternatives in treatment algorithms.

In conclusion, prophylactic low‐dose TCA, along with opioid and cannabis cessation, may be important strategies in the management of CVS.

## Author Contributions

M.F.B. wrote all versions of this manuscript and took the lead in data analysis. C.S. and F.C. were involved in data collection. A.D. assisted in data analysis. M.C. supervised the project. All authors read and approved the final manuscript. M.F.B. presented these data in an oral presentation at the British Society of Gastroenterology Annual Conference (Liverpool, UK) 2024.

## Conflicts of Interest

The authors declare no conflicts of interest.

## Data Availability

The data that support the findings of this study are available from the corresponding author upon reasonable request.
